# A case report of delirium tremens in a patient with myasthenia gravis

**DOI:** 10.1192/j.eurpsy.2025.1458

**Published:** 2025-08-26

**Authors:** V. Palinić, M. Krešić Ćorić, N. Zovko

**Affiliations:** 1 Psychiatry, University Clinical Hospital Mostar, Mostar, Bosnia and Herzegovina

## Abstract

**Introduction:**

This report describes a case of delirium tremens in a 35-year-old male patient with myasthenia gravis and epilepsy. He first came to our clinic on 18^th^ of August 2024. His complaints were of bad mood, nervousness and loss of will and appetite, he started to drink alcohol. He was advised to quit drinking and was prescribed quetiapine.

**Objectives:**

On 20^th^ of August he was hospitalised in our clinic as he presented with the withdrawal state. That, along with his initial blood tests, indicated a high probability of developing delirium tremens and presented us with the problem of treating this patient given his pre-existing condition.

The patient was started on saline with vitamins and glucose infusions. Low doses of quetiapine (25-50 mg) were given.

Current literature regarding MG and medications, puts diazepam in risk level 4, which means it rarely worsens MG, but symptoms must be monitored. Considering the high doses of diazepam usually required in states of delirium tremens and also not being able to predict how the strain of the delirious state will affect a patient with MG, we were cautious in administrating benzodiazepines.

**Methods:**

A neurologist was consulted and they evaluated there was no need to transfer the patient or to adjust his medication. His chronic therapy was pyridostigmine 3x60 mg and valproic acid 2x600 mg. Blood tests showed a rise in CK level from11463 U/L to 12793 U/L in just a few hours. Myoglobin was at 630 ng/mL. Potassium levels were initially 2.9 mmol/L and were corrected to the value of 3.8 in the control test. An internal medicine specialist was called for but did not come that day. The patient was more agitated, began hallucinating, profusely sweating and trembling trough out the night. He was physically restrained.

The following day, blood test showed a further increase of CK (15578 U/L). His general state was progressively worsening, and we resorted to administering low doses of diazepam and haloperidol along quetiapine.

On Aug 22^nd^, test results were: urea 6.9 mmol/L, creatinin 112 umol/L, K 4.0 mmol/L, Na 149 mmol/L, Cl 111 mmol/L, CRP 91.3 mg/L, AST 381 U/L, ALT 211 U/L, LDH 653 U/L, CK 17747 U/L, CKMB 134 U/L, Mg 0.72 mmol/L, amonia 58.28 umol/L. The patient was transferred to an IC unit where he was treated for two days and during which time he was respiratory stable but in need of constant electrolyte and pH correction. His CK levels were as high as 43400 U/L and myoglobin 5542.8 ng/mL.

**Results:**

On August 24^th^ the patient was returned to our clinic to continue his treatment. He was hospitalised for 20 more days and released home in good health.

**Conclusions:**

in emergency psychiatry there are sometimes cases which exceed our competences and practical possibilities to treat, but because they’re psychiatric in origin, those patient are admitted in psychiatric hospitals. There is need for therapeutic guidelines for patients who develop delirium tremens and are burdened with comorbidities.

**Disclosure of Interest:**

None Declared

